# Carcinoma

**Published:** 1866-12

**Authors:** A. Given

**Affiliations:** Louisville, Ky.


ARTICLE XLV.

### CARCINOMA.—DEATH FROM ULCERATION AND PERFORATION OF THE STOMACH WITHOUT TENDERNESS OR PAIN.

BY A. GIVEN, M.D., Louisville, Ky.

Mr. J. C., aged 63 years, was the oldest pilot on the Ohio River, and a man of robust constitution, weighing 260 pounds, previous to his last illness. During his last trip up the river, the latter part of February, 1866, he was taken with remittent fever, which, after arriving in this city, assumed a typhoid form with thin and watery stools. By the 1st of April, he was convalescing, diarrhœa checked, and appetite returned. He then went into the country to spend a few weeks, and, while there, his bowels became costive and refused to act, although some of the most powerful cathartics had been used. He now began to vomit. An enema was given, which procured a large stool. The injections had to be repeated from time to time to insure an operation, for the most active cathartics had no effect on the bowels. Since his death, which occurred on the 25th of September, I received a letter from Dr. G. H. HALL, of Bitter Water, Ky., in which he says:—

"I saw Mr. J. C. on the 14th of April, 1866, and found the following symptoms:—want of appetite, amounting almost to anorexia; obstinate constipation; conjunctiva of a yellowish tinge; inability to sleep; and a peculiar tingling sensation and formication in the forearms. There were no symptoms, and, certainly, at that time, no physical signs indicative of serious abnormal condition of the liver. There was not even the slightest uneasiness in any part of the right or left hypochondrium or in the epigastrium; he never complained a particle of his stomach, nor was there at that time or afterwards, while under my treatment, a single symptom, save the character of the fluids ejected from the stomach, to sustain a diagnosis of organic disease of that viscus; yet, everything indicated the utmost torpor of all the chylopoietic viscera, unattended, however, by the slightest uneasiness. There was great obscurity in the case. I advised him to return to the city, as he could not recover."

After returning to the city, he was treated by two physicians for habitual constipation and dyspepsia. I saw him, for the first time, on the 24th of July, and found him in the following condition:—Pulse 80 per minute and regular; tongue covered with a white fur, and moist; stomach and bowels slightly distended; appetite good; sleeps well; bowels had not moved for eight days; had acid eructations, and vomited every two or three days; there was no nausea or straining to vomit. I ordered an enema to be given, which procured a moderate-sized stool, with a large amount of flatus. I then examined the abdomen carefully, but found no pain or tenderness and no enlargement of the internal viscera. The eyes looked a little jaundiced. The absence of pain, tenderness, fever, and the character of the vomiting left the diagnostic symptoms very obscure. Being pressed for my opinion, I stated it to be a case of chronic inflammation of the stomach and duodenum, with a thickening of the mucous membrane, and, probably, ulceration of one or both. With this view of the case, I applied my treatment accordingly. The vomiting and eructations were checked in a few days and three healthy stools (one on each alternate day) procured without cathartics or injections, after giving the second injection. He gained strength, so as to be able to leave the house, and continued to improve until the 27th of August, when he began to vomit again and complained of a slight burning sensation in the œsophagus and a tingling sensation of the soles of the feet and arms, spreading up to his thighs, shoulders, and sides of the tongue. His appetite and strength now began to fail. I gave an injection, but failed to procure an evacuation. Thinking that there might be an obstruction in the sigmoid flexure of the colon, I passed a tube high into the bowel and injected, but failed to procure any fæces. I then came to the conclusion that there had but little passed the duodenum. As he was very feeble, and medicine had no effect, I discontinued medicines and ordered him to have any nourishment he desired, and, as he was a habitual drinker, to allow him some weak brandy. He took no nourishment for five weeks, except a little coffee and bread. He had no evacuation from his bowels for five weeks, until the day previous to his death, when he had a healthy-looking stool. His pulse was regular up to this time, when it became slower and slower, until he died without a pain or struggle. He retained his mental faculties until the last. The last time I spoke to him, I asked him if he suffered any; he replied that he was perfectly easy.

The manner of his death and the character of his symptoms made it an interesting case. It was evident that he died from inanition, but the degree of morbid action in the alimentary canal sufficient to cut off or impair the great function of assimilation could not be determined by the outward symptoms. I obtained permission, and made a *post mortem* examination, assisted by Drs. SUTTON, RONALD, and FOREMAN. We found the liver pale; the gall-bladder distended; the intestinal canal was empty and contracted; the duodenum and pyloric end of the stomach had a contracted feel; two inches from the pylorus, in the lesser curvature, the stomach was perforated. I laid open the stomach, and found the mucous membrane unhealthy and spotted; around the perforation the tissues were softened and easily broken down; near the pylorus, we found the tissues softened, ulcerated, and of a honeycomb appearance, exuding a thick, creamy pus; on the anterior wall, near the lesser curvature, we found an ulcer, oval in shape, two inches in diameter, with thickened borders, a concave, grayish, and indurated surface; at the pyloric pouch, we found a sinus, under which the peritoneal coat of the stomach (an inch in diameter) was thin and brown; the mucous membrane of the duodenum was reddened and thickened, so much so as to prevent the flow of bile through the duct, thereby causing the distension of the gall-bladder. The condition of the alimentary canal generally, and the stomach in particular, led us to suppose that the case before us was one of chronic inflammation and thickening of the tissues of the stomach, caused by excessive use of ardent spirits and high living; and arriving at that point when artificial stimulants could no longer arouse vital affinity of the parts, disintegration, softening, ulceration, and perforation of the stomach was the result. But we were, reluctantly, driven from this position by the microscope. Dr. J. W. BENSON, Prof. of Clinical Surgery in the University of Louisville, examined the stomach under the microscope, but failed to discover the cancer cells in the pus or internal tissues, but in the juice taken from a granular mass on the external coat, the cancer cells were very distinct.

The interesting points in this case are:—1st. The obscurity of the case, mildness of the symptoms, and the manner of his death. 2d. Was it a true hereditary cancer, whose germ lay dormant for 63 years in the system of one of the most robust and healthy men to be found, and aroused to action by alcohol; or was it created by the morbid action of the tissues, and nursed by intemperate habits until a nucleus was formed, around which the disease was fully developed, independent of a hereditary diathesis?

